# Necrotic cone photoreceptor cell death in retinitis pigmentosa

**DOI:** 10.1038/cddis.2015.385

**Published:** 2015-12-31

**Authors:** Y Murakami, Y Ikeda, S Nakatake, J W Miller, D G Vavvas, K H Sonoda, T Ishibashi

**Affiliations:** 1 Department of Ophthalmology, Graduate School of Medical Sciences, Kyushu University, Fukuoka, Japan; 2 Retina Service, Massachusetts Eye and Ear Infirmary, Department of Ophthalmology, Harvard Medical School, Boston, MA, USA

Retinitis pigmentosa (RP) comprises a group of inherited retinal degenerations, resulting from rod and cone photoreceptor cell death. Genetic studies have identified mutations in more than 50 genes—most of which encode rod-related molecules—that are associated with RP. Although rod cells that harbor the deleterious mutations are expected to die, it remains a puzzle why cone cells also die in this disease, subsequent to rod degeneration. Because the loss of cone-mediated vision is the most debilitating aspect of RP, elucidating the mechanisms of cone cell death is critical towards developing novel therapeutics in RP.

Apoptosis and necrosis are two major forms of cell death, which show distinct morphological appearance. Apoptosis is accompanied by the reduction of cellular volume and chromatin condensation, and necrosis is associated with cellular and organelle swelling, and plasma membrane rupture. Although necrosis was traditionally considered as an unregulated form of cell death, it is now known to have regulated components, such as those involving receptor-interacting protein (RIP) kinases.^1^

In animal models of RP, rod cell death has been shown to occur mainly through apoptosis.^2^ Recent studies demonstrated that caspase-independent pathways, such as poly-ADP-ribose-polymerase, calpain and histone deacetylase, are commonly activated in dying rod cells in several models of RP.^3, 4^ In contrast, the mode of cone cell death is less characterized. We and others previously demonstrated that, in mouse models of RP, cone cell death is associated with necrotic features and it is substantially suppressed by RIP3 deficiency or the RIP kinase inhibitor.^5, 6^ These findings indicated that the necrotic pathway is involved in cone cell death in RP, at least in part, and may be a novel therapeutic target (Figure 1). However, the relevancy of these findings in human pathology remains unclear.

In our recent study published in *Cell Death and Discovery*, we investigated the possible involvement of necrotic cone cell death in RP patients by examining the cone mosaic images obtained by the adaptive optics scanning laser ophthalmoscopy (AO-SLO).^7^ With the AO imaging system, the individual cone cells are visualized as bright spots in living human eyes. Using an automated measurement program of the cone spot size in AO-SLO images, we showed that there was a population of enlarged cone spots in the macula of RP patients. Cone enlargement was observed in a variety of RP patients and disease stage. The precise interpretation of these changes in AO-SLO images is challenging, however, taken together with the results from experimental studies, we propose that the enlarged spots may reflect the necrotic changes of cone cells in RP patients. Consistent with this idea, previous histological studies of postmortem RP patients' eyes demonstrated the necrotic changes of cone cells such as cell swelling and disrupted plasma membrane.^8^

Intracellular contents released from dying or dead cells act as damage-associated molecular patterns (DAMPs) to promote inflammatory responses and tissue injury. HMGB1 is one of the best-characterized DAMPs released from necrotic cells.^9^ In the vitreous of RP patients, we found that the HMGB1 levels were significantly elevated compared with those in controls. These findings support our idea that necrotic cell death is implicated in the degenerative process of RP. In animal models of RP, we previously demonstrated that RIP3 deficiency suppresses microglial activation during cone but not rod degeneration in rd10 mice, suggesting that DAMPs released from necrotic cone cells may enhance retinal inflammation.^5^ In human RP patients, intraocular inflammation measured by slit-lamp or laser flaremeter is inversely associated with central visual function.^10^ These findings indicate that cone cell death and inflammation are closely interconnected during cone degeneration in RP.

Recent studies have shown that the cone cell death in RP is caused by the microenvironmental changes following rod degeneration, such as inflammation, oxidation and loss of trophic factors. Interestingly, Punzo *et al.*^11^ reported that, in four different models of RP the dying cones show gene expression profiles associated with starvation. In line with these findings, Ait-Ali *et al.*^12^ recently showed that rod-derived cone viability factor promotes cone cell survival by stimulating the glucose uptake and glycolysis. Because nutrient deprivation can lead to mitochondrial and cytoplasmic swelling, the necrotic cone cell death in RP may be caused by a nutrient shortage. Alternatively, as inflammatory cells express tumor necrosis factor-α and FAS ligand, which recruit RIP1 and activate RIP kinase pathways, excessive inflammatory activation may exacerbate necrotic cone cell death in RP. It will be interesting to further investigate the mechanisms of cone cell death in several models and human patients with RP to identify the common therapeutic targets to prevent or delay the secondary cone degeneration in RP.

## Figures and Tables

**Figure 1:**
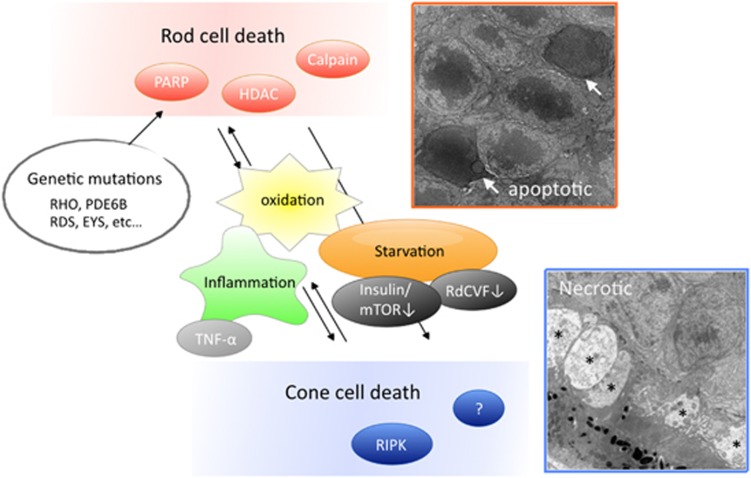
Rod and cone photoreceptor cell death in retinitis pigmentosa. Rod cell death due to the deleterious genetic mutations is associated with apoptosis, which involves the activation of caspase-independent pathways including poly-ADP-ribose-polymerase (PARP), calpain and histone deacetylase (HDAC). Cone cell death is induced by the microenviromental changes subsequent to rod degeneration, such as oxidation, inflammation and loss of trophic factors. Dying cones show different morphological features from rod cells, such as necrotic cytoplasmic swelling (asterisk), and is partly mediated through the activation of RIP kinase (RIPK). Electron microscopy images were reproduced with permissions from Murakami *et al.*^5^ mToR, mammalian target of rapamycin; RdCVF, rod-derived cone viability factor; TNF-α, tumor necrosis factor-α
